# *Fusarium graminearum *forms mycotoxin producing infection structures on wheat

**DOI:** 10.1186/1471-2229-11-110

**Published:** 2011-07-28

**Authors:** Marike J Boenisch, Wilhelm Schäfer

**Affiliations:** 1 Biocenter Klein Flottbek, Molecular Phytopathology and Genetics, University of Hamburg, Ohnhorststrasse 18, 22609 Hamburg, Germany

## Abstract

**Background:**

The mycotoxin producing fungal pathogen *Fusarium graminearum *is the causal agent of Fusarium head blight (FHB) of small grain cereals in fields worldwide. Although *F. graminearum *is highly investigated by means of molecular genetics, detailed studies about hyphal development during initial infection stages are rare. In addition, the role of mycotoxins during initial infection stages of FHB is still unknown. Therefore, we investigated the infection strategy of the fungus on different floral organs of wheat (*Triticum aestivum *L.) under real time conditions by constitutive expression of the *dsRed *reporter gene in a *TRI5prom*::*GFP *mutant. Additionally, trichothecene induction during infection was visualised with a green fluorescent protein (GFP) coupled *TRI5 *promoter. A tissue specific infection pattern and *TRI5 *induction were tested by using different floral organs of wheat. Through combination of bioimaging and electron microscopy infection structures were identified and characterised. In addition, the role of trichothecene production for initial infection was elucidated by a Δ*TRI5*-*GFP *reporter strain.

**Results:**

The present investigation demonstrates the formation of foot structures and compound appressoria by *F. graminearum*. All infection structures developed from epiphytic runner hyphae. Compound appressoria including lobate appressoria and infection cushions were observed on inoculated caryopses, paleas, lemmas, and glumes of susceptible and resistant wheat cultivars. A specific trichothecene induction in infection structures was demonstrated by different imaging techniques. Interestingly, a Δ*TRI5*-*GFP *mutant formed the same infection structures and exhibited a similar symptom development compared to the wild type and the *TRI5prom*::*GFP *mutant.

**Conclusions:**

The different specialised infection structures of *F. graminearum *on wheat florets, as described in this study, indicate that the penetration strategy of this fungus is far more complex than postulated to date. We show that trichothecene biosynthesis is specifically induced in infection structures, but is neither necessary for their development nor for formation of primary symptoms on wheat.

## Background

*Fusarium graminearum *Schwabe (teleomorph *Gibberella zeae *(Schwein) Petch) is the main causal agent of Fusarium head blight (FHB) disease of small grain cereals and cob rot of maize [1-3]. Mycotoxins produced by *Fusarium *species result in a loss of yield and reduced quality of grains [4-6]. Fusarium toxins including the trichothecenes nivalenol (NIV), deoxynivalenol (DON) and its derivatives 3- and 15-acetyldeoxynivalenol (3-ADON, 15-ADON) contaminate cereal products and have been shown to be harmful to humans, animals, and plants [4]. Hence, the European Union and the United States set limits for DON in final products for human consumption of 0.75 μg/g [Commission Regulation [EC] no. 1881/2006] and of 1 μg/g [Council for Agricultural Science and Technology, 2003]. Trichothecenes are potent phytotoxins for many plants. They can produce symptoms including wilting, chlorosis and necrosis at concentrations of 10^-5 ^to 10^-6 ^M [7]. The toxic effect of trichothecenes is mainly due to their ability to bind to the 60S ribosomal subunit of eukaryotes, resulting in inhibition of protein synthesis and induction of apoptosis [8]. DON is a virulence factor in wheat [9-14]. DON production enables the fungus to spread from infected florets into the wheat rachis [9-13].

Although detailed information of the *TRI *gene cluster is currently available [15], the factors inducing mycotoxin production during infection on wheat are unknown. The first step of the trichothecene biosynthesis is catalysed by the enzyme trichodiene synthase encoded by the *TRI5 *gene [5,16]. Thus, the *TRI5 *gene is used as a marker gene for the induction of trichothecene biosynthesis of the fungus [17-20]. Induction assays using axenic cultures revealed *TRI5 *inducing conditions and substances under laboratory conditions [17,18,20-22]. However, little is known about *TRI5 *inducing factors *in planta*. Wheat infection of a fungal mutant with a *TRI5 *promoter GFP (green fluorescent protein) fusion revealed a tissue specific *TRI5 *induction [20]. A high *TRI5 *induction in the rachis node and in caryopses was observed. *GFP *and *TRI5 *expression were much lower in the adjacent rachis and no GFP fluorescence was observed on anthers, although they were heavily colonised. One objective of the current study was to test different parts of the infected wheat spikelet for tissue specific infection patterns and *TRI5 *induction. *F. graminearum *is one of the most intensively studied fungal plant pathogens [3,6,23], but our knowledge about fungal development on host surfaces and the penetration strategy of the pathogen during initial infection stages is limited. Thus, another objective of this study was to investigate fungal development and the penetration strategy of *F. graminearum *by different bioimaging techniques including bright field images, fluorescence microscopy, confocal laser scanning microscopy (CLSM), and scanning electron microscopy (SEM). We performed an *in vitro *bioassay that allows a continuous microscopic evaluation of detached glumes, lemmas, paleas, and caryopses under comparable conditions. The *in vitro *bioassay was designed according to Jansen et al. [12], who performed a similar assay to investigate caryopses of wheat and barley infected with *F. graminearum *reporter strains. In addition, an *in vitro *bioassay using previously frozen, detached glumes for microscopic studies of initial infection of different *F. graminearum *mutants was recently published [24]. In the present study, monitoring of the infection process and *TRI5 *induction in real time was possible by use of reporter strains. We achieved a continuous observation of fungal development *in planta *by constitutive expression of the *dsRed *gene from *Discosoma *spec.. The expression of *GFP *gene driven by the endogenous *TRI5 *promoter in the reporter strain enabled monitoring of the trichothecene induction during infection in real time. Previous studies demonstrated that *TRI5prom*::*GFP *exhibits wild type-like growth and infectivity, *TRI5 *expression and DON production [20]. Infection structures and *TRI5 *induction were studied on different floret organs of wheat. In the field, FHB disease is initiated by airborne spores landing on flowering spikelets [3,25,26]. Open florets during anthesis provide opportunities for the pathogen to contact primary penetration sites. Developing caryopses as well as the adaxial surfaces of lemma and palea are only accessible in open florets and are comparably susceptible to infection [3,27,28]. *F. graminearum *initially colonises the surface of wheat florets without immediate penetration [28,29]. Most *Fusarium *species enter husks of wheat and barley by natural openings, such as stomata [3,29-32], or penetrate epidermal cell walls with short infection hyphae [23,28,31,33-36]. *F. graminearum *is described as a pathogen that does not form different types of appressoria [23,34-36]. However, several recent publications provide light microscopy images of lobed, highly septate, and corralloid hyphal structures, which might be involved in penetration of glumes [24,30,32]. These authors distinguish between subcuticular coral-like hyphal mats and bulbous infection hyphae. The mitogen-activated protein kinase (MAPK) mutants Δ*gpmk1 *[37,38] and Δ*mgv1 *[39], and also the GTPase Δ*ras2 *mutant [36], were tested for their ability to form coral-like subcuticular structures and bulbous infection hyphae. While Δ*mgv1 *and Δ*ras2 *mutants were able to form coralloid, subcuticular hyphae and bulbous invasive hyphae similar to those of the wild type strain PH-1, the Δ*gpmk1 *mutant formed coralloid subcuticular hyphae but no bulbous hyphae. Thus, *gpmk1 *gene of *F. graminearum *was discussed to be involved in the formation of bulbous infection hyphae. Additionally, first evidence was provided that papillae silica cells are preferred sites for invasion. We describe specific infection structures of *F. graminearum *wild type isolate 8/1 and reporter strains with 8/1 background. By combination of bioimaging and SEM on floret tissues infected with a *TRI5prom*::*GFP *strain and a trichothecene deficient Δ*TRI5-GFP *strain, the role of trichothecenes for different initial infection stages was evaluated. Comprehensive imaging techniques together with molecular analyses might improve our knowledge about early plant-pathogen interactions during FHB infection.

## Results

### Screening of infection and *TRI5 *induction in real time

**Figure 1:**
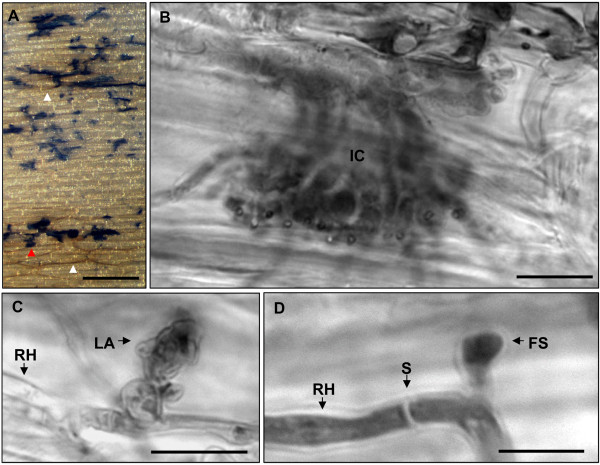
**Infection structures of *F. graminearum *wild type isolate 8/1 on wheat cv Nandu**. **A-D** Light microscopy of infected palea after trypan blue staining of mycelium at 10 dpi. **A** Abundance of infection cushions (blue) on the surface of palea under white light conditions using MZFLIII microscope. Different sizes of infection cushions and necroses of plant cells (white arrowhead) surrounding big infection cushions is remarkable, scale bar = 200 μm. **B-D** Higher magnification using Zeiss Axio Imager.Z1 reveals different infection structures. **B** Magnification of blue stained fungal structure (red arrowhead in A) showing a typical infection cushion, scale bar = 50 μm. **C** Typical cellular structure of a lobate appressorium and **D** foot structure arising from runner hyphae, scale bars = 10 μm. **Abbreviations: FS** Foot structures, **IC** infection cushions, **LA** lobate appressorium, **RH** runner hypha, **S** septum.

**Figure 2:**
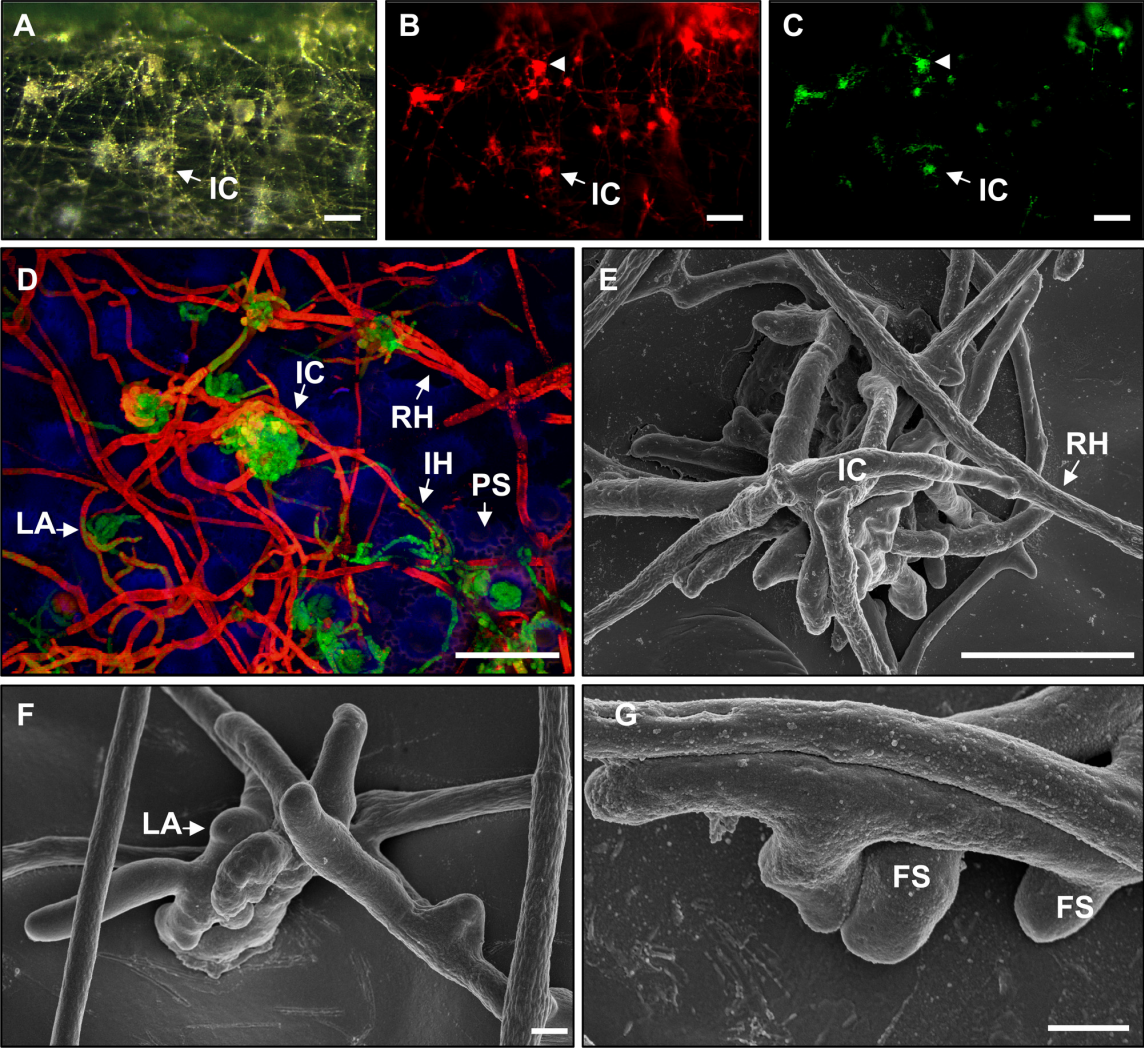
**Infection structures and *TRI5 *induction of *F. graminearum TRI5prom::GFP *on wheat cv Nandu**. **A-C **White light and fluorescence micrographs of infection cushions on palea at 8 dpi using MZFLIII microscope, scale bars = 100 μm. **A **Natural appearance of the inoculated surface of palea. **B **Infection cushions are visible by dsRed fluorescence. **C **GFP fluorescence demonstrates *TRI5 *induction in infection structures. **D **Laser scanning microscopy of GFP inductive fungal structures (white arrowhead in B and C). Overlay image of individually detected dsRed and GFP fluorescence of the fungus as well as blue plant autofluorescence. The image represents a maximum intensity projection of a z-stack, scale bar = 50 μm. **E-G **Scanning electron micrographs of different infection structures on glume at 8 dpi. **E **Infection cushion, scale bar = 50 μm. **F **Lobate appressorium, and **G **foot structures, scale bars = 2 μm. **Abbreviations: FS **Foot structures, **IC **infection cushion, **IH **infection hypha, **LA **lobate appressorium, **PS **papillae silica cell, **RH **runner hyphae.

We observed similar stages of infection on all tested floral organs infected with the wild type 8/1 and the *TRI5prom*::*GFP *strains using a MZFLIII microscope. The process of infection could be divided into three stages (Table 1). Stage I: Initial colonisation stage. Within 6-12 h germination took place and the hyphal growth spread over the surface. Hyphal growth was not notably directed to topographical features like longitudinal junctions between epidermal cells. Short infection hyphae indicate direct penetration during stage I. However, no disease symptoms were apparent at this stage. Homogenous hyphal networks were usually formed on caryopses until 1-2 days after inoculation, on paleas until 4-5 dpi and until 6-7 dpi on lemmas and glumes. From the respective time points onward hyphal morphology became heterogeneous. Stage II: Main infection stage. During this stage runner hyphae began to branch at a high frequency and formed foot structures, lobate appressoria and infection cushions. Figure 1 shows the typical stage II of a wild type infection using light microscopy. Removal of chlorophyll from husk tissues and trypan blue staining of hyphae revealed necrotic plant cells surrounding infection cushions (Figure 1A). These lesions appeared at the earliest at 3 dpi on caryopses, at 5 dpi on paleas and at 7 dpi on glumes and lemmas. Higher magnifications (Figure 1B-D) revealed cytological similarities to described infection cushions of other fungal phytopathogens [40-50]. Large infection cushions were formed by highly branched and agglomerated hyphae (Figure 1B). Additionally, smaller infection structures similar to lobate appressoria (Figure 1C) and foot structures (Figure 1D) were observed, as described for *Rhizoctonia solani *[40,42,43,51-53]. Trichothecenes are potent phytotoxins that can evoke symptoms like necrosis in a wide variety of plants at very low concentrations [7]. We considered that trichothecenes might be involved in the development of necrotic lesions on wheat tissues. Therefore, we performed the *in vitro *bioassay with conidia of a *TRI5prom*::*GFP *mutant. A specific *TRI5 *induction was observed in infection cushions and not in the runner hyphae as identified by studies combining light microscopy using MZFLIII microscope (Figure 2A-C) and confocal laser scanning microscopy (CLSM) (Figure 2D). Infection hyphae, lobate appressoria, and infection cushions showed an induction of GFP on all inoculated organs (data not shown). Furthermore, CLMS revealed direct penetration of epidermal cells through GFP inductive infection structures (Additional file 1). Interestingly, the GFP inductive structures were visible especially in papillae silica cells (Additional file 1). Epifluorescence microscopy revealed infection cushions increased in sizes during the following 1-2 days, while GFP induction decreased (data not shown). Disease symptoms such as brownish necrosis, as well as chlorosis develop rapidly in the ongoing infection stage. Stage III: Final infection stage. The entire tissue of husks is necrotic, and aerial hyphae as well as sporodochia are produced. Infection stages I-III occurred likewise at the resistant wheat cultivar Sumai 3 (Additional file 2) and the susceptible cultivar Nandu (Figure 1 and 2). Therefore, formation of infection structures and GFP induction was not affected by the susceptibility or resistance of the cultivar. To test whether formation of compound appressoria and GFP induction was dependent on the inoculation method with detached floral organs, we examined floret organs of intact plants after spikelet point inoculation as a control. Infection stages I-III were observed on husks of the susceptible cultivar Nandu and the semi-resistant cultivar Amaretto within 7 dpi of investigation. Conidia germinated within the first 6-12 hpi. Between 3 and 4 dpi GFP inductive infection cushions developed on caryopses, paleas, and lemmas (Additional file 3) demonstrating stage II. From 4 dpi onward many aerial hyphae were formed and between 5 and 7 dpi tissues were entirely necrotic and chlorotic. In contrast to the other organs, glumes were not colonised within 7 dpi. No differences were observed between the wheat cultivars Nandu and Amaretto. Investigations using spikelts on intact wheat plants (n = 2) were repeated three times with both cultivars with similar results.

**Table 1:** Infection stages I-III on floret organs of wheat and their omnipresent characteristics

	Stage I	Stage II	Stage III
**Main event**	Surface colonisation	Penetration	Sporulation

**Typical fungal morphology**	Runner hyphae Infection hyphae	Runner hyphae Foot structures Lobate appressoria Infection cushions	Aerial hyphae Sporodochia

**DON-Induction**	No	Yes	No

**Necroses**	No	Some	Many

Observations were made at glumes, lemmas, paleas, and caryopses of the susceptible wheat cultivar Nandu (n = 80/organ) and the resistant cultivar Sumai 3 (n = 24/organ) infected with *TRI5prom::GFP *mutant in detached *in vitro *bioassays.

### SEM of infection structures

Ultrastructural characterisation of GFP inductive infection structures was performed by combination of fluorescence microscopy and SEM. We identified GFP inductive infection cushions (15-50 μm in diameter) (Figure 2E), lobate appressoria (5-15 μm in diameter) (Figure 2F), and foot structures (4-5 μm in diameter) (Figure 2G). After removal of infection cushions from glumes, SEM revealed numerous penetration pores of about 1-2 μm in diameter in the epidermal cell walls (data not shown).

### DON quantification in wheat floret tissues

**Figure 3:**
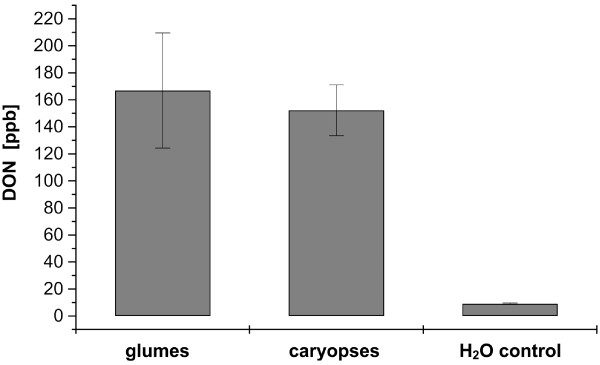
**Quantification of deoxynivalenol (DON) in glumes and caryopses infected with *F. graminearum TRI5prom::GFP *at 8 dpi**. 28 infected glumes and 28 caryopses of the wheat cultivar Nandu were pooled for the estimation of DON after GFP-fluorescence of the fungus was visible by fluorescence microscope MZFLIII. The concentration of DON in ppb of infected fresh weight was estimated by the RIDASCREEN DON enzymatic immunoassay (R-Biopharm, Darmstadt, Germany). Three replicative measurements were done for statistical analyses. Mock inoculated glumes were used as a negative control.

We measured the amount of DON in glumes and caryopses using an enzyme-linked immunosorbent assay for DON detection to confirm that optical detection of GFP fluorescence of *TRI5prom*::*GFP *reporter strain results in a detectable production of trichothecenes. The results revealed high DON amounts in glumes and caryopses (167 ± 43 ppb and 152 ± 19 ppb) (Figure 3). There is no detectable difference between the DON amount in glumes and caryopses (difference 15 ppb) considering the minimum detection limit of 18.5 ppb for the used method to quantify DON and the standard errors of the measurements of ± 43 ppb for glumes and ± 19 ppb for caryopses. The results demonstrate that the used *TRI5prom*::*GFP *reporter strain produced high amounts of DON after GFP fluorescence was visually detected during plant infection.

### Initial infection of a DON deficient mutant

**Figure 4:**
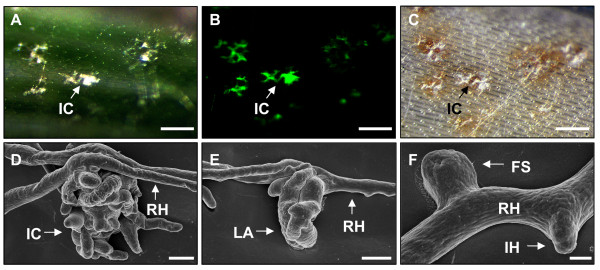
**Infection structures of a trichothecene deficient Δ*TRI5-GFP *mutant of *F. graminearum *on wheat cv Nandu**. **A-C **White light and fluorescence micrographs of an infected glume at 13 dpi done with MZFLIII microscope, scale bars = 200 μm. **A **White light image of infection cushions formed by the Δ*TRI5-GFP *mutant. **B **GFP fluorescence of the mycelium due to constitutive GFP expression. **C **Brownish necroses around infection cushions visible by white light microscopy after critical point drying. **D-F **Scanning electron micrographs show different infection structures of a Δ*TRI5-GFP *mutant on glume at 8 dpi. **D **Infection cushions, **E **lobate appressoria, **F **foot structures and infection hyphae, scale bars: D and E = 4 μm, F = 1 μm. **Abbreviations: FS **Foot structure, **IC **infection cushion, **IH **infection hypha, **LA **lobate appressorium, **RH **runner hypha.

To evaluate the role of trichothecenes for initial penetration, we investigated infections using a trichothecene deficient *TRI5 *knockout strain (Δ*TRI5*-GFP) with constitutive *GFP *expression. Microscopic investigations revealed no differences between the early infection stages of Δ*TRI5*-*GFP *mutant (Figure 4), and the *TRI5prom*::*GFP *reporter strain (Figure 2). Necrotic lesions around infection cushions were observed by light microscopy using a MZFLIII microscope (Figure 4A-C). Necrotic plant cells were visible after removal of chlorophyll by critical point drying (Figure 4C). They refer to plant destruction at fungal penetration sites. SEM of glumes infected by Δ*TRI5*-*GFP *mutant revealed that all types of infection structures including infection cushions (Figure 4D), lobate appressoria (Figure 4E), foot structures and infection hyphae (Figure 4F) were formed by the Δ*TRI5-GFP *mutant. In summary, DON production occurs specifically in infection structures, but is neither required for their formation nor for formation of necrotic lesions in their surroundings.

In the following (Table 2), we summarise the presented experiments and microscopic methods.

**Table 2:** Overview of experiments and microscopic methods

Microscopy of infection	Detached *in vitro *assay	Intact *in vivo *assay
**Cultivar/strain combinations (Figure no.)**	Nandu/WT (1) Nandu/*TRI5prom::GFP *(2) Nandu/Δ*TRI5-GFP *(4) Sumai 3/*TRI5prom::GFP *(2*)	Nandu/*TRI5prom::GFP *(3*) Amaretto/*TRI5prom::GFP *(3*)

**Time points of MZFLIII screening**	all cultivar/strain combinations 6 hpi - 14 dpi (1 A, 2 A-C, 4 A-C, 2 A-C*, 3 A-F*)	6 hpi - 7 dpi (3 A-F*)

**Microscopy with Axio Imager Z1**	all cultivar/strain combinations (2* D)	Nandu/*TRI5prom::GFP* Amaretto/*TRI5prom::GFP *(3 G*)

**CLSM**	Nandu/*TRI5prom::GFP *(2 D, 1*)	-

**SEM**	Nandu/*TRI5prom::GFP *(2 E-F) Nandu/Δ*TRI5-GFP *(4 D-F)	-

The different cultivar/strain combinations, investigated time of infection and the microscopic methods used *in vitro *and *in vivo *bioassays are detailed. Respective figure numbers are given in brackets (Additional File*).

## Discussion

### Infection stages and infection structures of *F. graminearum *on wheat

The colonisation of the inside of the wheat floret tissues by *F. graminearum *was evaluated in detail [12,20,28,29,54,55], but information about fungal development on different wheat tissues and the penetration strategy of the fungus is limited. Three successive infection stages (stage I-III) were distinguishable on all floret tissues.

We did not observe disease symptoms during stage I. This is consistent with previous findings that hyphae of *F. graminearum *initially branch symptomlessly on the exterior surfaces of floret tissues from wheat and barley, and do not penetrate the epidermis immediately after germination [6,29,31]. The disease symptoms of spelts and caryopses as well as sporodochia production described for stage III confirm earlier descriptions of late infection stages of FHB on floret wheat tissues [6,12,32]. A very surprising result was the formation of compound appressoria during stage II of infection. Lobate appressoria and infection cushions are two types of so called compound appressoria [44]. They are described as multicellular types of appressoria, formed by irregularly shaped hyphae [44]. *F. graminearum *develops compound appressoria with striking morphological similarities to other fungal plant pathogens e.g. *Rhizoctonia solani *[40,42,48,52,53], *Botrytis cinerea *[46,49], and *Sclerotinia sclerotiorum *[44,47]. Previous publications provided the first microscopic evidences for more complex infection structures formed by *F. graminearum *[24,30,32]. Two different structures were described, namely coral-like hyphal mats [24,30,32] and bulbous infection hyphae [24]. Coral-like hyphal structures were further specified by lobed, thickened, and branched hyphae [24,30,32]. We suggest that the coral-like hyphal mats are infection cushions, whereas the bulbous infection hyphae correlate to foot structures. However, coral-like infection structures were observed already at 24 hpi on glumes, while infection cushions appeared on glumes at 7 dpi in our assays. The difference might be explained by strain specific variations between the European wild type isolate Fg 8/1 we used, and the American strain PH1. Furthermore, the investigators studying PH1 used detached glumes, which were stored frozen before inoculation. In contrast to fresh detached glumes as used in our studies, the defence reactions of refrozen plant cells might be strongly reduced.

*F. graminearum *is commonly described to enter floret tissues of cereals by direct penetration via infection hyphae or by natural openings, such as stomata [14,29,30,32]. Penetration through stomata by lobate appressoria and infection cushions was observed only occasionally during stage II, but seems to be undirected. The earliest infection structures observed are infection hyphae. Short infection hyphae are described in detail for different *Fusarium *species by ultrastructural studies using TEM and SEM [27,28,33,56]. In our studies, infection hyphae were visible on all wheat organs during stage I and II. We were not able to appraise the number of actually penetrating infection hyphae during stage I. Thus, the impact of infection hyphae for further disease progression is not elucidated. Interestingly, the tissues remained macroscopically asymptomatic until necrotic lesions surrounding infection cushions appeared during stage II. Necrotic lesions on cotton hypocotyls infected with *R. solani *appeared when infection cushions were fully developed [40]. This is consistent with our observations that lesions develop mainly around bigger infection cushions (Figure 1A and 4C).

Compound appressoria were formed on different types of tissues, like caryopses and spelts, but, depending on the type of organ, at different time points after inoculation (data not shown). In summary, the results demonstrate that compound appressoria are formed independently of the biological function and morphological characteristics of different organs. They were formed on vegetative organs with silicified epidermal cells, but also on the pericarp of the caryopsis without silica cells. Silicified epidermal cells seem to be a preferred site of penetration, but are not a prerequisite of successful infection. Developing caryopses are highly susceptible for infection due to the absence for cell wall thickenings in the pericarp and underlying tissues. Additionally, the results exclude a dependence of compound appressoria formation on certain topographical features, because the epidermal architecture of bracts and caryopses is fundamentally different. Topographical features of the inoculated surface [40], the availability of nutrients [40,57] and plant exudates [40,43,57-59] were discussed to be influencing factors for development and morphology of compound appressoria from *R. solani*. Several abiotic factors which influence fungal growth and development, like humidity, temperature, exposure to light, and nutrient availability will probably influence the development of compound appressoria. For example, exposure to light has been shown to influence infection cushion formation of *R. solani *[40]. We propose that foot structures, lobate appressoria, and infection cushions mirror different developmental stages of infection cushion formation, similar to *R. solani *[40,50]. A schematic model that illustrates the development of infection cushions by *R. solani *is provided by Armentrout and Downer [40]. Furthermore, a development of lobate appressoria into infection cushions is described for *R. solani *[60], *B. cinerea *[49], and *S. sclerotiorum *[44]. The increasing size and complexity of cellular structures observed during the time of infection supports the idea of a developmental process. It is currently unknown whether infection hyphae of *F. graminearum*, observed during stage I are involved in the development of compound appressoria.

In comparison to infection cushions, lobate appressoria are smaller and formed by fewer cells. They can be formed by a single lobate cell, but two-celled to multi-celled types are described as well [44]. In general, hyphae of lobate appressoria are short, swollen, and highly septate [44]. Each lobe of a lobate appressorium can form an infection peg [44]. The development of compound appressoria from epiphytic mycelium is common for several other phytopathogenic fungi [44]. Direct penetration of epidermal cells by infection hyphae, infection cushions, and lobate appressoria was evident by CLSM (Figure 2D and Additional file 1). The presence of penetration pores in the outer epidermal cell wall underneath infection cushions provides a hint for numerous penetration pegs. Many penetration pores resulting from penetration pegs of infection cushions were demonstrated by comparable ultrastructural studies on *S. sclerotiorum *[47] and *R. solani *[45,53]. The primary hyphae from which compound appressoria originate are termed runner or running hyphae in other infection cushion producing fungi such as *R. solani *[45,50], several *Sclerotinia *species [41,47,61] and *Gaeumannomyces graminis*, the causal agent of take-all patch disease [62,63]. The observed runner hyphae are clearly distinguishable from other functionally specified hyphae, e.g. hyphae that form infection structures, aerial hyphae or reproductive hyphae (e.g. conidiophores and ascogenic hyphae).

The early infection process of susceptible and resistant wheat cultivars (Additional file 2 and 3) were indistinguishable. Infection structure formation as well as DON induction occurred comparably. This is consistent with the fact that type II resistance of Sumai 3 and Amaretto may prevent systemic infection of the wheat spikes, but does not prevent initial infection of the inoculated wheat spikelet [64-66]. An artificial effect on compound appressoria formation due to the dissection of floral organs was ruled out by microscopic evaluation of inoculated spikelets from intact potted wheat plants. The investigation demonstrated infection cushion formation on the surface of paleas, lemmas, and caryopses (Additional file 3). Glumes were not colonised by hyphae and showed no infection cushions at 4 dpi. The difference in colonisation between glumes and adaxial surfaces of paleas, lemmas, and on caryopses might depend on the applied inoculation method. Injecting conidia inside the wheat floret provides no initial contact of conidia to the glume surface. In bioassays using detached wheat tissues, infection cushions were formed later compared to tissues of intact plants. This might be explained by differences in humidity, light quality, and temperature differences. Adding additional nutritions, i.e. sugars, to the detached flower leaf assays resulted in much shorter infection times. Nevertheless, all different infection structures were formed at 2-3 dpi (data not shown).

### Infection structures and DON induction

Previous infection studies with *TRI5 *knockout mutants on wheat revealed that the trichothecene deficient mutant is still able to infect the inoculated wheat spikelet like wild type [9,11-13]. Therefore, trichothecenes seem to be of minor importance for initial infection stages on wheat. In contrast, a major role of trichothecenes for the initial establishment in the host tissue was suggested [19], because induction of *TRI5 *expression was detected during early infection stages on barley spikes infected with *F. graminearum *[67] as well as on seedlings of wheat infected with *F. culmorum *[19]. A tissue specific induction of the *TRI5 *pathway during spike infections was indicated recently [20]. In the present study we demonstrated that *TRI5 *expression is specifically induced in infection structures. The detection of high amounts of DON in glumes and caryopses infected with *TRI5prom*::*GFP *(Figure 3) confirmed that visible GFP fluorescence correlates to DON production of the mutant. Interestingly, infection structures and necrotic lesions are formed independently of trichothecene production. Several explanations seem possible. Firstly, the lesions surrounding the infection cushions are caused by the multiple penetration pores. These cellular destructions might induce host defence responses, similar to plant reactions by wounding (e.g. oxidative burst, apoptosis, modifications of the plant cell wall). Secondly, virulence factors, like an already described secreted lipase [68] and most likely other not yet discovered factors, act independently of DON, cause the observed destruction, and are responsible for the initial infection. The question remains why DON is induced, even though it does not contribute to the virulence of the fungus at this time of infection. DON is necessary to suppress plant defence enabling the pathogen to break through the rachis node. DON production is strongly induced, most likely by the host, at this point of infection [12,20]. It seems possible that similar host factors induce DON at other stages of infection, for example during the penetration of the cuticle. This might explain why a DON deficient mutant colonises maize cobs similar to wild type, although the wild type produced high levels of DON during the infection [13].

Different host compounds can be discussed to be responsible for *TRI5 *induction during infection. Nitrogen and carbon sources as well as low pH play key roles in regulation of mycotoxins produced by *Aspergillus *species, such as sterigmatocystin and aflatoxin [69,70]. Many nitrogen containing substances like various amines were identified that significantly induce *TRI5 *expression [17]. The amine putrecine induced *TRI5 *expression and mycotoxin production *in vitro *to levels observed during infection [17]. In addition, it was demonstrated that low extracellular pH is required for DON production in axenic culture [17]. A combination of low pH and amines results in significantly enhanced expression of the *TRI5 *gene and increased DON production. The factors responsible for *TRI5 *induction of infection structures *in planta *has to be evaluated in further investigations.

## Conclusions

We provided here the first evidence that *F. graminearum *is able to form lobate appressoria and infection cushions during FHB infection. It is generally believed that *Fusarium *species invade host tissues without generating an appressorium [23,24,34,35]. In the present study we demonstrated that the penetration strategy of *F. graminearum *on wheat florets is more complex than postulated until now. Compound appressoria were formed on different types of tissues like caryopses and husks. Therefore, a tissue independent formation of compound appressoria is demonstrated. A major role of cushion formation on symptom development is suggested by necrotic lesions and penetration pores underneath cushions. A specific induction of trichothecenes in infection structures was demonstrated by CLSM. Consequently, a relation between DON production and direct penetration was investigated. Interestingly, infections with a trichothecene deficient mutant revealed no differences compared to infections with trichothecene producing strains. Trichothecene biosynthesis is specifically induced in infection structures, but not a prerequisite for their development and the initial penetration of wheat tissues. In summary, the combination of different bioimaging techniques with functional reporter strains and electron microscopy provided new insights into the penetration strategy of *F. graminearum *and the role of trichothecene induction during initial infection of wheat. A detailed knowledge about early development of FHB might help to explore new ways of disease control.

## Methods

### Plant material

The susceptible wheat cultivar Nandu (Lochow-Petkus, Bergen-Wohlde, Germany), the resistant Chinese wheat cultivar Sumai 3 and the semi-resistant cultivar Amaretto (B. Bauer, Niedertraubling, Germany, FHB resistance category 3) were grown in the greenhouse in plastic pots at 18-20°C, 60% relative humidity, and a photoperiod of 16 h.

### Fungal material and preparation of inoculum

To investigate the initial stages of the infection on wheat florets we used a previously described *TRI5prom*::*GFP *reporter mutant [20]. Localisation of the mycelium was possible due to constitutive expression of the *dsRed *gene under control of the glycerol-3-phosphate dehydrogenase (*gpdA*) promoter of *Aspergillus nidulans*. To evaluate the induction of DON during infection, the green fluorescence protein (*GFP*) gene was fused to the promoter of the *TRI5 *gene, coding for the trichodiene synthase of the fungus. Mutants were generated as described previously [13]. The resulting *TRI5prom*::*GFP *mutant consists of a fully functional *TRI5 *gene and exhibits GFP fluorescence driven by an endogenous *TRI5 *promoter [20]. The second reporter strain used (Δ*TRI5-GFP*) is DON deficient and expresses *GFP *constitutively under the control of *gpdA *promoter, which enables the detection of mycelium during infection under real time conditions [12]. Both *F. graminearum *reporter strains were generated by transformation of the wild type isolate Fg 8/1 [71].

Vegetative conidia of *F. graminearum *strains were obtained as described previously [13]. The concentration of conidia suspension was adjusted to 2 × 10 ^4 ^conidia/ml and stored at -70°C.

### Detached *in vitro *bioassay

For the detached *in vitro *bioassay, spikelets of three-month-old wheat plants were taken at anthesis to isolate floret organs including caryopses, paleas, lemmas and glumes. Organs were detached from the floret with a razor blade and placed in Petri dishes (92 × 16 mm, SARSTEDT) on 1.6% (w/v) water agar (Difco granulated agar; Becton Dickinson). Four Petri dishes contained 8 biological replicates of one floret organ and represented one independent experiment. The ventral side of caryopses and the adaxial side of glumes, lemmas and paleas were inoculated with 5 μl sterile water containing 2 × 10^4 ^conidia/ml. After inoculation, the Petri dishes were sealed with Parafilm (Pechiney, Chicago, USA) and incubated in a growth chamber at 16 h light period with 18°C and 16°C at darkness. Floret organs of the susceptible cultivar Nandu and the resistant Chinese wheat cultivar Sumai 3 were inoculated equally with the *TRI5prom*::*GFP *strain to test for cultivar dependent differences during infection. Floret tissues of Sumai 3 plants were inoculated with 2.5 μl of a 4 × 10^4 ^conidia/ml water suspension. Conidia of a Δ*TRI5-GFP *mutant were used for inoculation to study infections of floret organs under trichothecene deficient conditions. Floret organs of the susceptible cultivar Nandu infected with conidia of the wild type isolate Fg 8/1 served as a control for the wild type-like character of observations derived from the *TRI5prom*::*GFP *strain.

### Intact *in vivo *bioassay

To test for artificial effects due to wounding and detachment from the plant in the *in vitro *bioassay, intact spikelets on potted wheat plants inoculated with macroconidia of *TRI5prom*::*GFP *were investigated microscopically. Spike inoculations were performed with three-month-old wheat plants at anthesis [13]. 10 μl of conidia suspension containing 2 × 10^4 ^conidia/ml were injected into the cavity between lemma and palea. The incubation took place in a growth chamber under similar conditions as described for detached *in vitro *assay. The susceptible cultivar Nandu and the semi-resistant cultivar Amaretto were investigated equally.

### Monitoring of *TRI5 *induction during infection

The infection was studied in a two step process. Firstly, we screened infected organs using the MZFLIII microscope. Thereby fungal development, *TRI5 *induction and disease symptoms of the entire surface of the same sample were studied from conidia germination until sporulation. The typical stages of infection and *TRI5 *induction on different floret tissues were identified by the screening with MZFLIII. In the second step, we characterised *TRI5 *inductive fungal structures in detail by epifluorescence microscopy, CLSM, and SEM.

### Screening of infection

Organs infected with the *TRI5prom*::*GFP *strain in *in vitro *bioassays were monitored with a MZFLIII microscope (see below) at 6, 12, and 24 hpi. From 24 hpi onward investigations proceeded in 1 day intervals until 14 dpi. Ten independent inoculation experiments (n = 8) were performed with organs of the cultivar Nandu infected with the *TRI5prom*::*GFP *strain provided similar results. The role of trichothecenes for the initial infection was evaluated in *in vitro *bioassays by using a Δ*TRI5-GFP *mutant for inoculation of floret organs of the cultivar Nandu. The colonisation of the host and plant necrosis was investigated with MZFLIII microscope at given above time points from 6 hpi to 14 dpi. Wheat organs of the resistant cultivar Sumai 3 were infected with the *TRI5prom*::*GFP *strain and investigated for cultivar dependent differences in colonisation, *TRI5 *induction, and plant necroses at time points given above. Floret organs infected with *TRI5prom*::*GFP *strain from attached *in vivo *assays were examined at a one day interval from 3 dpi to 7 dpi. We used white light and fluorescence microscopy of MZFLIII microscope to examine the colonisation, *TRI5 *induction and plant necroses. *In vivo *assays of the intact spikelet of potted wheat plants (n = 2) were repeated three times with similar results. Floret organs of the susceptible cultivar Nandu infected with conidia of the wild type isolate Fg 8/1 were investigated with MZFLIII microscope at given time points from 6 hpi to 14 dpi. Three independent experiments (n = 8) were performed with floral tissues of Sumai 3 infected with the *TRI5prom*::*GFP *strain and floral tissues of the cultivar Nandu infected with Fg 8/1 or the Δ*TRI5-GFP *mutant.

### MZFLIII light microscopy

Infected floret organs were investigated with MZFLIII microscope (Leica Microsystems, Heerbrugg, Switzerland) in air without preparation, lying in Petri dishes. Inclined reflected light of an external halogen lamp KL 1500 Electronic (Schott, Mainz, Germany) was used to visualise plant necroses as well as the mycelium under white light conditions with Leica MZFLIII microscope. The dsRed fluorescence of the *TRI5prom*::*GFP *strain was detected with the Leica dsRed filter set containing an excitation filter at 546/12 nm and a long pass filter at 560 nm. The GFP fluorescence of the *TRI5prom*::*GFP *and Δ*TRI5-GFP *strain was visible by Leica GFP filter set with an excitation filter at 470/40 nm and a band pass filter transmitting light at 525/50 nm. To investigate the infection of the *F. graminearum *wild type 8/1, plant tissues were incubated in 96% ethanol for fixation and removal of chlorophyll. The mycelium was stained with 0.1% (w/v) trypan blue solution (Fluka analytical) in 10% (v/v) acetic acid for 20 minutes at room temperature. Excessive stain was removed by rinsing samples with distilled water. Samples were cut in pieces and transferred on glass slides for white light images of infected tissues. The Leica MZFLIII fluorescence microscope was equipped with a Leica 1.0 × objective. Photos were done with Leica DFC 500 coloured camera. The LAS Leica software (version 2.7.1) was used for image acquisition and procession.

### Microscopy of infection structures

#### Bright field and epifluorescence microscopy

To investigate infection structures of the *F. graminearum *wild type 8/1 we used bright field microscopy with transmitted light of a Zeiss Axio Imager.Z1 microscope. Infection structures of reporter strains were investigated by fluorescence microscopy using Zeiss Axio Imager.Z1 microscope equipped with a Zeiss Apotome. A UV (ultra violet) lamp HAL 100 served as UV light source. DsRed was excited in the range of 538 to 562 nm and detected in the 570 to 640 nm range. GFP was excited with 450 to 490 nm and detected at 500 to 550 nm wavelength. The plant apoplast was excited in the range of 335 to 383 nm. The blue autofluorescence was detected in the 420 to 470 nm range. Images were taken with Zeiss AxioCam MRm CCD camera. Image processing, including overlay of different fluorescence channels and generation of maximum intensity projections (MIP) of z-stacks were done with Zeiss AxioVision software (version 4.8.1).

#### Laser scanning microscopy

To investigate the penetration of the epidermal layer through compound appressoria of *F. graminearum *laser scanning microscopy was performed. Paleas and glumes infected with *TRI5prom*::*GFP *were analysed with Leica TCS SPE microscope (Leica Microsystems, Wetzlar, Germany). DsRed was excited at 532 nm and detected in the 560 to 683 nm range. GFP was excited at 488 nm and detected in the 520 to 631 nm range. Autofluorescence of the plant apoplast was excited at 405 nm and detected in the 408 nm to 464 nm range. The sample shown in Figure 2D and Additional file 1 represents an area of 275 × 275 μm in xy-dimension. 22.47 μm were scanned by 107 steps in z-dimension. Image processing, maximum projection, and data analyses were done with the Leica LAS AF software.

#### Scanning electron microscopy

Four glumes from detached *in vitro *bioassays infected with *TRI5prom*::*GFP *or Δ*TRI5-GFP *were sampled each at 8 dpi. The glumes were fixed with 4% (v/v) glutaraldehyde in 50 mM phosphate buffer (pH 6.8) for 8-10 h at 4°C, then rinsed with the same buffer for 3 h. Afterwards, samples were post-fixed with 1% (w/v) osmium tetroxide in the same buffer for 2 h at 4°C. After dehydration for 24 h by a graded acetone series at room temperature, the samples were critical-point dried, mounted on stubs, and sputter-coated with gold. The scanning electron microscope SEM LEO 1525 was used operating at 6 kV.

### DON quantification in wheat floret tissues

To confirm that an optical detection of GFP fluorescence of *TRI5prom*::*GFP *reporter strain resulted in a production of trichothecenes, the concentration of DON in ppb (parts per billion) of fresh weight of infected glumes and caryopses was quantified after GFP fluorescence of infected samples was observed by fluorescence microscopy using MZFLIII microscope. DON measurements were carried out by using the RIDASCREEN DON enzymatic immunoassay kit (R-Biopharm, Darmstadt, Germany). 28 infected glumes and caryopses were pooled and weighed at 8 dpi. Water inoculated glumes at 8 dpi were used as negative control. Pooled samples were pestled in liquid nitrogen and homogenates were diluted 1:5 (w/v) with sterile water according to respective fresh weight. Samples had been shaken for 30 min at 1500 rpm and 25°C in the thermomixer comfort (Eppendorf, Hamburg, Germany) for DON extraction. By centrifugation for 10 minutes at 25°C and 3500 rpm in the centrifuge MiniSpin (Eppendorf, Hamburg, Germany), the supernatant was freed from cell debris. 50 μl of the respective extract was used in the DON assay. The following experimental procedure, measurement and calculation were done according to RIDASCREEN DON kit manual. The extinction was measured by photometry at 450 nm by a 96-well-ELISA-reader Anthos 2010 (Mikrosysteme GmbH, Krefeld, Germany). The measurements were repeated three times for statistical analyses. The DON- concentration of the extracts was estimated by a calibration curve with DON standards. Calculations, statistics and graphics were done by using Microcal Origin calculation software (version 5.0).

## Authors' contributions

MJB carried out all the experimental work and drafted the manuscript. WS coordinated the experimental work, was involved in analysis and interpretation of data as well as in drafting and revising of the manuscript. Both authors read and approved the final manuscript.

## Supplementary Material

Additional file 1 **GFP inductive fungal structures penetrate epidermal cells**. CLSM image of infected palea shown in Figure 2D. The video contains 170 single images of the z-stack with 22 μm thickness in total. The z-series starts at the top of the biggest infection cushion (centre of image) and ends in the second or third cell layer underneath the epidermal layer. Fungal infection structures can be observed from the surface into the plant tissue. Tangential optical sections of the epidermis demonstrate that GFP inductive infection cushions and lobate appressoria invade epidermal cells. Click here for file

Additional file 2 **Infection structures and *TRI5 *induction on resistant cv Sumai 3, inoculated with *F. graminearum TRI5prom::GFP *at 12 dpi**. **A-C **White light and fluorescence microscopy with MZFLIII microscope, scale bars = 200 μm. **A **Surface of glume shows infection cushions and runner hyphae. **B **Due to dsRed fluorescence runner hyphae and infection cushions are visible. **C **Infection cushions but no runner hyphae show high GFP fluorescence. **D **Overlay image of dsRed and GFP fluorescence of the fungus as well as blue plant autofluorescence detected with Zeiss Axio Imager.Z1. The picture is a maximum intensity projection of a z-stack that demonstrates GFP fluorescence of infection cushions, scale bar = 100 μm. **Abbreviations: BIC **Big infection cushion, **ECW **epidermal cell wall, **IC **infection cushion, **PS **papillae silica cell, **RH **runner hypha, **SIC **small infection cushion. Click here for file

Additional file 3 **Infection and *TRI5 *induction after inoculation of intact wheat plants with *F. graminearum TRI5prom::GFP***. **A-G **Infection stage II of different floret tissues of infected spikelets of the susceptible cv Nandu (**A-C**) and the medium resistant cv Amaretto (**D-G**) at 4 dpi. **A-F **White light and fluorescence micrographs of infected palea (**A-C**) and caryopsis (**D-F**) were done using MZFLIII microscope. **A **and **D **Weak disease symptoms are visible. **B **and **E **Infection cushions are identified by dsRed fluorescence. **C **and **F **Infection cushions show high GFP induction. **G **Overlay image of GFP fluorescence and blue plant autofluorescence detected with Zeiss Axio Imager. Z1. GFP inductive infection cushions on the adaxial surface of lemma are demonstrated. The picture is a maximum intensity projection of a z-stack. Scale bars: A-C = 1 mm, D-F = 500 μm and G = 200 μm. **Abbreviations: IC **Infection cushion, **T **trichome. Click here for file
