# Supplementary figures and images for: Fusarium graminearum forms mycotoxin producing infection structures on wheat
Source: BMC Plant Biol. 2011 Jul 28;11:110. doi: 10.1186/1471-2229-11-110 (PMC3166921; doi:10.1186/1471-2229-11-110)

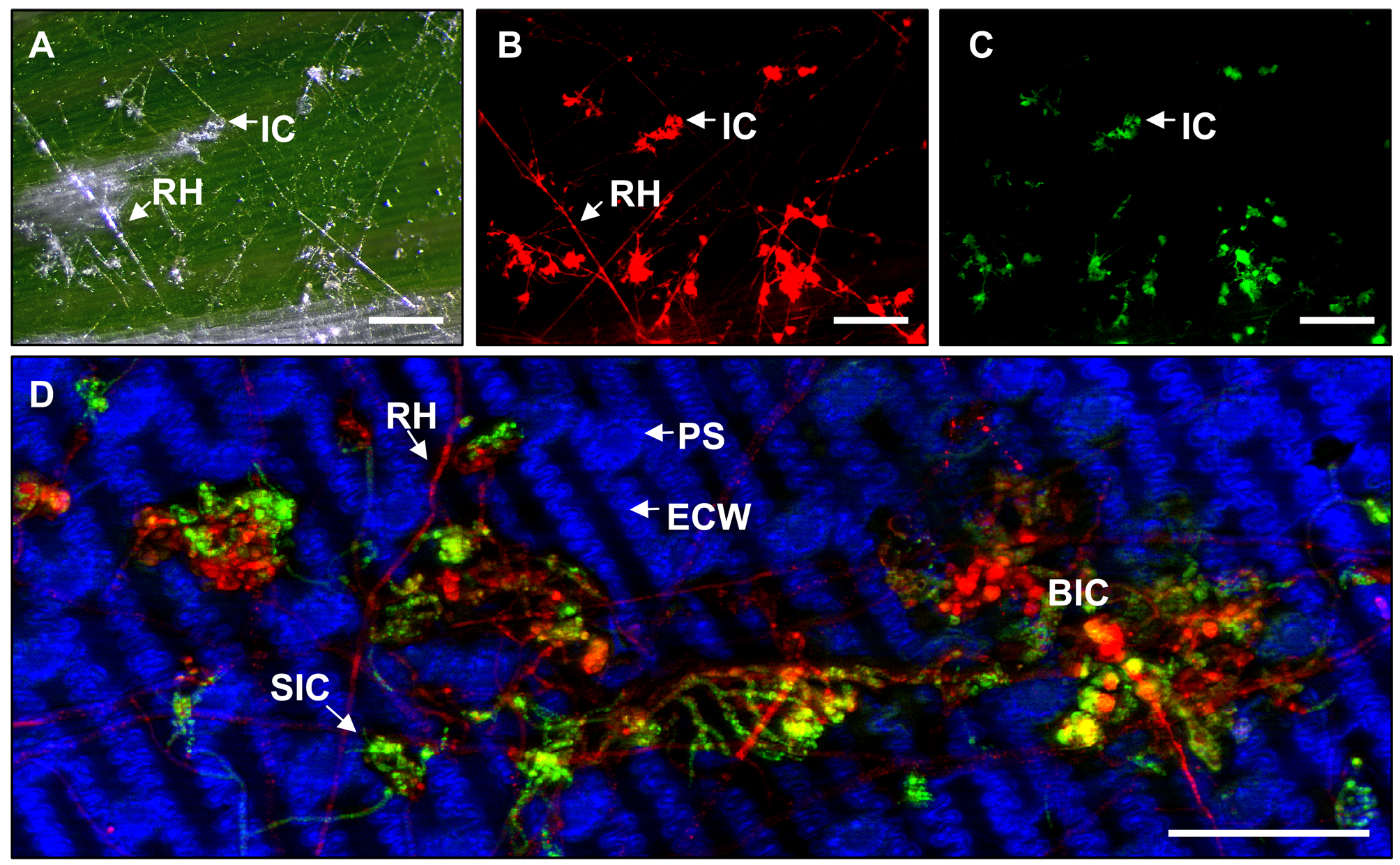

Supplement: Additional file 2 — Infection structures and TRI5 induction on resistant cv Sumai 3, inoculated with F. graminearum TRI5prom::GFP at 12 dpi. A-C White light and fluorescence microscopy with MZFLIII microscope, scale bars = 200 μm. A Surface of glume shows infection cushions and runner hyphae. B Due to dsRed fluorescence runner hyphae and infection cushions are visible. C Infection cushions but no runner hyphae show high GFP fluorescence. D Overlay image of dsRed and GFP fluorescence of the fungus as well as blue plant autofluorescence detected with Zeiss Axio Imager.Z1. The picture is a maximum intensity projection of a z-stack that demonstrates GFP fluorescence of infection cushions, scale bar = 100 μm. Abbreviations: BIC Big infection cushion, ECW epidermal cell wall, IC infection cushion, PS papillae silica cell, RH runner hypha, SIC small infection cushion. [file 1471-2229-11-110-S2.TIFF]

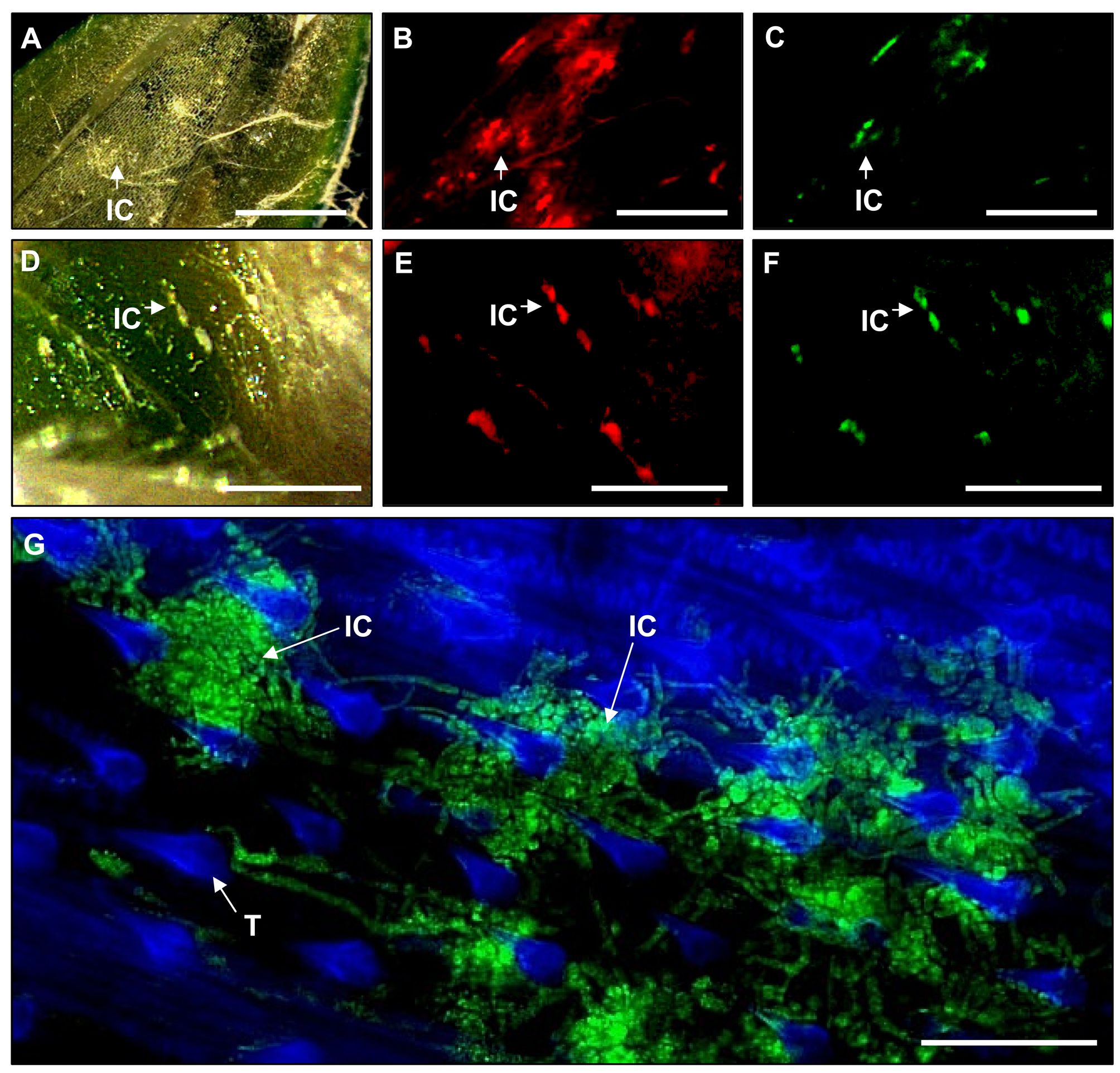

Supplement: Additional file 3 — Infection and TRI5 induction after inoculation of intact wheat plants with F. graminearum TRI5prom::GFP. A-G Infection stage II of different floret tissues of infected spikelets of the susceptible cv Nandu (A-C) and the medium resistant cv Amaretto (D-G) at 4 dpi. A-F White light and fluorescence micrographs of infected palea (A-C) and caryopsis (D-F) were done using MZFLIII microscope. A and D Weak disease symptoms are visible. B and E Infection cushions are identified by dsRed fluorescence. C and F Infection cushions show high GFP induction. G Overlay image of GFP fluorescence and blue plant autofluorescence detected with Zeiss Axio Imager. Z1. GFP inductive infection cushions on the adaxial surface of lemma are demonstrated. The picture is a maximum intensity projection of a z-stack. Scale bars: A-C = 1 mm, D-F = 500 μm and G = 200 μm. Abbreviations: IC Infection cushion, T trichome. [file 1471-2229-11-110-S3.TIFF]
